# Prevalence and Associated Factors of Gastroesophageal Reflux Disease After Laparoscopic Sleeve Gastrectomy

**DOI:** 10.7759/cureus.57921

**Published:** 2024-04-09

**Authors:** Maather M Abdulkhaleq, Reema S Alshugaig, Dania A farhan, Ibtihal t Balubaid, Rahaf A Alkhaldi, Fatema m Shoaib, Fatmah m Shamaa, Saleh M Aldaqal

**Affiliations:** 1 Faculty of Medicine, King Abdulaziz University Faculty of Medicine, Jeddah, SAU; 2 General Surgery, King Abdulaziz University Faculty of Medicine, Jeddah, SAU

**Keywords:** #laparoscopic sleeve gastrectomy, gastroesophageal reflux disease (gerd), obesity, prevalence of gerd, gerd-hrql

## Abstract

Objectives

To determine the prevalence of gastroesophageal reflux disease (GERD) after laparoscopic sleeve gastrectomy (LSG) and associated factors.

Methods

A cross-sectional study was conducted in different regions around the Kingdom of Saudi Arabia between 2022 and 2023. The questionnaire was distributed among patients who underwent LSG at different periods, ranging from six months to more than two years. The questionnaire comprised a risk factor assessment and the GERD-Health-Related Quality of Life (GERD-HRQL) questionnaire.

Results

A total of 387 participants with a mean age of 35.7±10.95 were included. The study included 225 females (58.1%) and 162 males (41.9%). The mean preoperative body mass index (BMI) was 44.36±8.07 kg/m^2^, which decreased to 28.78±6.31 kg/m^2^ postoperatively. Notably, dissatisfaction with general health surged from 17 (24.6%) preoperatively to 165 (42.6%) postoperatively. Despite no significant difference in GERD-HRQL scores in the group who had preoperative symptoms, 282 (72.9%) reported experiencing heartburn, and 289 (74.7%) reported bloating postoperatively. Postoperatively, 203 (52.5%) reported improved quality of life. Moreover, changes in BMI were strongly correlated with heartburn, dysphagia, odynophagia, and bloating. The postoperative prevalence of GERD was 355 (91.7%), with 318 (82.2%) of participants reporting new-onset symptoms. Sex (P=0.013), age (P=0.024), and hypercholesterolemia (P=0.046) were significantly associated with postoperative GERD severity.

Conclusions

The majority of participants developed GERD symptoms following surgery, with a significant proportion reporting new-onset symptoms. Sex, age, and hypercholesterolemia have emerged as significant factors for postoperative GERD severity.

## Introduction

Obesity is a devastating public health concern in developed countries, influencing both genders of all ethnicities and socioeconomic backgrounds [1]. According to various epidemiological studies, one in every five individuals is considered obese worldwide, with the Middle East exhibiting the highest prevalence rates of obesity [2]. Reported obesity rates among Saudi adults have ranged from 13% to 70% over the past six decades [3]. Moreover, obesity has been linked to several preventable co-morbidities and health outcomes, including diabetes, cancer, heart disease, and obstructive sleep apnea syndrome [4]. Gastroesophageal reflux disease (GERD) is a multifactorial disorder commonly observed in obese patients due to chronically elevated intra-abdominal pressure, resulting in acidic gastric juices flowing backward into the esophagus and causing heartburn or regurgitation [5]. Bariatric surgery stands as the sole viable approach for obese patients to achieve substantial weight loss and a significant reduction of obesity-related co-morbidities over time [6]. Correspondingly, between 1998 and 2003, there was a 10-fold increase in the overall number of bariatric surgeries worldwide [7]. Since the beginning of the twenty-first century, laparoscopic sleeve gastrectomy (LSG) has gained global popularity, including in the American Society for Metabolic and Bariatric Surgery (ASMBS), and is considered the primary bariatric procedure [8]. The procedure is widely performed due to the low incidence of morbidity and mortality, the relatively short learning curve, and the minimal physiological impact [9]. Although the procedure is universally approved, LSG might be linked with the development of GERD symptoms compared with other bariatric procedures [10,11]. Studies have reported conflicting results regarding this matter, with limited research describing the long-lasting impacts of LSG on GERD symptoms in the Middle East. Therefore, this study aimed to measure the prevalence of GERD after LSG and determine other associated factors.

## Materials and methods

Study design

A descriptive, cross-sectional study was conducted around the Kingdom of Saudi Arabia between 2022 and 2023.

Data collection

An online, self-administered questionnaire was distributed across multiple social media platforms in Saudi Arabia. The questionnaire included five parts: the first part was the agreement to be involved in the research; the second part was a yes-or-no question to make sure the operation was laparoscopic sleeve gastrectomy; and the third part was demographic data, including name, age, sex, marital status, nationality, residency, smoking status, co-morbidities, height, preoperative and postoperative weight, previous diagnosis of GERD, and when the operation was performed. The fourth and fifth parts were scales by Vic Velanovich to measure the quality of life for preoperative and postoperative GERD symptoms, respectively [12]. The scale is called the GERD-health-related quality of life (GERD-HRQL) and includes 11 questions about heartburn, dysphagia, bloating, or gas; consuming reflux medication and the effects of the drugs on daily life; and health satisfaction. Each question was translated into Arabic. The questions were scored on a scale from zero to five, with a maximum score of 50 indicating a lower quality of life (QOL). 

Selection of participants

We employed a non-probability purposive sampling technique to approach participants who had undergone LSG in Saudi Arabia with a pre-or postoperative body mass index (BMI) of > 30 kg/m2, with or without co-morbidities and aged between 18 and 60 years. We excluded patients under 18 or over 60 years of age with a preoperative BMI less than 30 kg/m2, missing data, or undergoing other or previous bariatric surgeries. This study received approval from the Research Ethical Committee of King Abdulaziz University under reference number 502-23.

Statistical analysis

IBM Corp. Released 2015. IBM SPSS Statistics for Windows, Version 23.0. Armonk, NY: IBM Corp. was used to evaluate and test the hypotheses. Simple cross-tabulations, frequency tables, and percentages. Means and standard deviations (STDs) were determined as part of the descriptive statistics. The link between two related groups was investigated using a paired-sample t-test. The mean values for both groups were assessed through an independent sample t-test, while Pearson's correlation coefficient was employed to assess the strength and direction of the association between the two variables. The relationship between the two variables was investigated using the chi-square test. The cut-off value for significance was set at p<0.05.

## Results

Demographics

A total of 387 participants with a mean age of 35.87±10.95 years were included in the study. Male patients comprised 162 (41.9%) of the population, and 225 (58.1%) were female patients. The majority of the participants were Saudi nationals (348, 89.9%), with smokers comprising (106, 27.4%). Approximately four-fifths of the participants (315, 81.4%) had co-morbidities, the most common of which were dyslipidemia (55, 14.2%) and hypertension (52, 13.4%). Furthermore, 225 (58.1%) of the participants had the surgery more than two years ago. Preoperatively, 318 (82.2%) of the patients were not diagnosed with GERD. The mean height was 166.30±10.20 cm. The mean preoperative BMI was 44.36±8.07 kg/m2, and the mean weight was 123.39±28.63 kg. The mean postoperative BMI was 28.78±6.31 kg/m2, and the mean weight was 79.74±19.46 kg. The mean change in BMI was 15.57±6.94 kg/m2. The mean weight change was 43.66±21.25 kg. Before the operation, 17 (24.6%) of the participants were dissatisfied with their general health, 48 (69.6%) were neutral, four (5.8%) were satisfied compared to post-operation, 165 (42.6%) were dissatisfied, 188 (48.6%) were neutral, and 34 (8.8%) were satisfied with their general health. More details are listed in Table 1.

**Table 1:** Demographics and characteristics of participants (n=387). GERD: gastroesophageal reflux disease, STD: standard deviation The data has been represented as N, %, Mean±STD.

		n	%
Gender	male	162	41.9
	female	225	58.1
Marital Status	married	225	58.1
	unmarried	162	41.9
Nationality	Saudi	348	89.9
	non-Saudi	39	10.1
Region	Makkah	245	63.3
	Other	142	36.7
Smoking	Smoker	106	27.4
	Non-smoker	253	65.4
	X-smoker	28	7.2
Hypertension	yes	52	13.4
Diabetes	yes	43	11.1
Hypercholesterolemia	yes	55	14.2
Asthma	yes	36	9.3
Hypothyroidism	yes	46	11.9
Sleep Apnea	yes	23	5.9
Hiatal Hernia	yes	30	7.8
Others	yes	30	7.8
When was LSG performed?	< 6 months	51	13.2
	6–12 months	53	13.7
	1–2 years	58	15.0
	> 2 years	225	58.1
Diagnosis of GERD preoperatively	no	318	82.2
	yes	69	17.8
Preoperative satisfaction of general health	satisfied	4	5.8
	neutral	48	69.6
	dissatisfied	17	24.6
Postoperative satisfaction of general health	satisfied	34	8.8
	neutral	188	48.6
	dissatisfied	165	42.6
(Age) (years) (mean ± STD)	35.87±10.95		
(height) (cm) (mean ± STD (	166.30±10.20		
(mean ± SD) (weight before surgery ((Kg)	123.39±28.63		
(mean ± SD) (weight after surgery) (Kg)	79.74±19.46		
weight change (mean ± STD)	43.66±21.25		
BMI before op (mean ± STD)	44.36±8.07		
BMI after op (mean ± STD)	28.78±6.31		
BMI change (mean ± STD)	15.57±6.94		

GERD-HRQL questionnaire score

**Figure 1:**
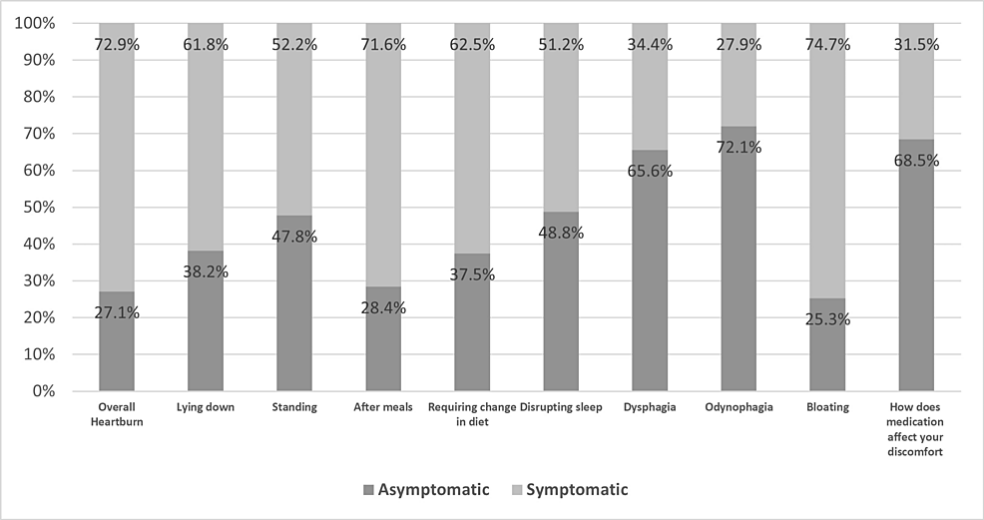
Postoperative prevalence of each GERD symptom for symptomatic and asymptomatic participants (n=387) The data has been represented as %.

The mean and STD scores for each GERD-HRQL questionnaire item pre- and postoperatively were not significantly different (Table 2). The mean postoperative heartburn score was 1.88, 1.64 for lying down, 1.20 for standing up, and 1.50 for sleep disturbance. Dysphagia and odynophagia had the lowest means of 0.87 vs. 0.64, respectively, and bloating and heartburn after meals had the highest means of 2.27 vs. 2.04, respectively (Table 3). Postoperatively, 32 (8.3%) had no symptoms of GERD, while 105 (27.1%) of patients experienced no heartburn but other symptoms of GERD. Additionally, 282 (72.9%) had overall heartburn, 239 (61.8%) when lying down, 202 (52.2%) while standing, 277 (71.6%) after having meals, 242 (62.5%) after diet changes, and 198 (51.2%) had sleep disturbances. The values for dysphagia and odynophagia were 133 (34.4%) and 108 (27.1%), respectively. Of the total patients, 289 (74.7%) experienced bloating, and 122 (31.5%) were consuming medication to reduce symptoms (Figure 1). No significant relationship was observed between preoperative BMI, weight, and GERD symptoms. In contrast, a significant correlation between BMI, weight, and BMI change after surgery concerning heartburn after meals, dysphagia, odynophagia, and bloating is demonstrated in Table 4.

**Table 2:** Comparison of symptoms before and after surgery in the study participants (n=69). GERD: gastroesophageal reflux disease, STD: standard deviation The data has been represented as Mean±STD and p-value. p<0.05 (significant)

GERD Symptoms	Before Operative n=69		After Operative n=69		p-value
	Mean	STD	Mean	STD	
Overall Heartburn	2.59	1.30	2.41	1.64	0.332
Lying down	2.46	1.60	2.19	1.70	0.110
Standing	1.67	1.49	1.81	1.49	0.392
After meals	2.71	1.64	2.49	1.60	0.178
Requiring change in diet	2.25	1.76	2.51	1.63	0.118
Disrupting sleep	2.19	1.92	2.13	1.89	0.732
Dysphagia	1.55	1.65	1.57	1.79	0.923
Odynophagia	1.16	1.50	1.29	1.60	0.388
Bloating	3.03	1.71	3.01	1.71	0.914
How does medication affect your discomfort?	1.59	1.83	1.86	1.83	0.198

**Table 3:** Postoperative symptoms of participants (n=387). GERD: gastroesophageal reflux disease, STD: standard deviation The data has been represented as Mean±STD.

GERD Symptoms	n=387	
	Mean	STD
Overall Heartburn	1.88	1.57
Lying down	1.64	1.69
Standing	1.20	1.41
After meals	2.04	1.77
Requiring change in diet	1.77	1.77
Disrupting sleep	1.50	1.83
Dysphagia	0.87	1.47
Odynophagia	0.64	1.24
Bloating	2.27	1.80
How does medication affect your discomfort?	0.90	1.53

**Table 4:** Correlation coefficient (r) and p-value of pre- and postoperative GERD symptoms and BMI, weight, and BMI change. GERD: gastroesophageal reflux disease, BMI: body mass index The data has been represented as (r)= Pearson's correlation coefficient and p-value. p<0.05 (significant), (r) is significant at the 0.01 level.

GERD Symptoms		Preoperative n=69		Postoperative n=387		
		Preoperative weight	BMI before op	Postoperative Weight	BMI after op	BMI change
Overall Heartburn	r	-0.075	0.059	-0.069	-0.075	-0.053
	p-value	0.538	0.629	0.175	0.143	0.297
Lying down	r	0.043	0.136	-0.048	-0.047	-0.060
	p-value	0.725	0.267	0.344	0.361	0.242
Standing	r	-0.088	0.057	-0.048	-0.036	0.002
	p-value	0.471	0.644	0.347	0.482	0.964
After meals	r	-0.210	-0.157	-.123-^*^	-.119-^*^	-0.087
	p-value	0.083	0.199	0.016	0.019	0.088
Requiring change in diet	r	0.029	0.113	-0.087	-0.074	-0.034
	p-value	0.813	0.355	0.086	0.146	0.500
Disrupting sleep	r	-0.027	0.053	-0.067	-0.056	-0.004
	p-value	0.827	0.664	0.190	0.268	0.933
Dysphagia	r	-0.153	-0.120	-0.044	-0.024	-.136-^**^
	p-value	0.210	0.325	0.389	0.635	0.007
Odynophagia	r	-0.009	0.028	-0.025	0.043	-.100-^*^
	p-value	0.941	0.818	0.621	0.399	0.049
Bloating	r	-0.039	0.034	-0.022	0.007	-.112-^*^
	p-value	0.750	0.780	0.664	0.884	0.028
How does medication affect your discomfort?	r	-0.018	0.043	-0.062	-0.004	-0.060
	p-value	0.883	0.723	0.226	0.930	0.243

The severity of GERD symptoms using GERD-HRQL

The heartburn score was determined by summing the individual scores for the 10 questions. The range, median, mean, and STD were determined to assess the severity. Moreover, preoperative and postoperative quality of life were categorized as poor or good according to the median values.

**Figure 2:**
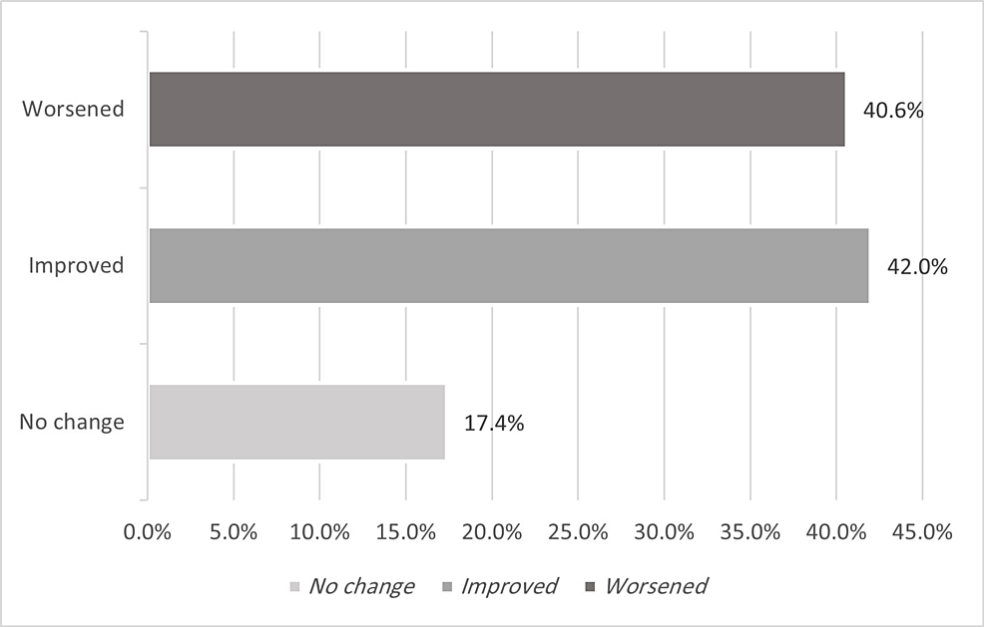
The proportion of participants whose symptoms changed after LSG (n=69) The data has been represented as %.

Therefore, we considered participants below or equal to the median as having "good QOL,” while those above the median were determined to have "poor QOL.” The GERD-HRQL scores ranged from 0 to 43 for the preoperative group, with a mean value of 21.20, an STD of 11.81, and a median score of 21. The postoperative group scores ranged from 0 to 50, with a mean of 14.72, an STD of 12.30, and a median score of 12. Preoperatively, of a total of 69 participants, 34 (49.3%) were identified as having "poor QOL," whereas 35 (50.7%) had "good QOL." Postoperatively, among 387 participants, 184 (47.5%) had poor QOL. In contrast, 203 (52.5%) had good QOL. Additionally, 29 (42%) of participants who developed GERD symptoms preoperatively displayed improvement in their symptoms after surgery; however, 28 (40.6%) of them had worsening symptoms, and 12 (17.4%) had a non-noticeable effect on their GERD score (Figure 2).

Characteristics and factors associated with GERD severity after surgery

We studied the relationship between some demographic characteristics and co-morbidities of the participants postoperatively and their GERD severity (Table 5). Among the 387 participants, we identified significant relationships between sex (p=0.013), age (p=0.024), and hypercholesterolemia (p=0.046). Moreover, 97 (59.9%) males demonstrated good QOL compared to 106 (47.1%) females. Young participants with a mean age of 34.68±11.22 displayed good QOL, while old participants with a mean age of 37.19±10.51 demonstrated poor QOL. Of the participants who did not have hypercholesterolemia, 181 (54.5%) displayed better QOL than 22 (40%). Smoking status, BMI, and other co-morbidities were not statistically significant (p > 0.05).

**Table 5:** Relationship between demographics, co-morbidities, and postoperative GERD score. GERD: gastroesophageal reflux disease, BMI: body mass index, QoL: quality of life. The data has been represented as N, %, p-value. p<0.05 (significant).

		Postoperative symptoms QOL score		Total	p-value
		poor	good		
Gender	male	65	97	162	0.013
		40.1%	59.9%	100.0%	
	female	119	106	225	
		52.9%	47.1%	100.0%	
smoking	Smoker	57	49	106	0.183
		53.8%	46.2%	100.0%	
	Non-smoker	117	136	253	
		46.2%	53.8%	100.0%	
	X-smoker	10	18	28	
		35.7%	64.3%	100.0%	
Hypertension	no	159	176	335	0.934
		47.5%	52.5%	100.0%	
	yes	25	27	52	
		48.1%	51.9%	100.0%	
Diabetes	no	164	180	344	0.886
		47.7%	52.3%	100.0%	
	yes	20	23	43	
		46.5%	53.5%	100.0%	
Hypercholesterolemia	no	151	181	332	0.046
		45.5%	54.5%	100.0%	
	yes	33	22	55	
		60.0%	40.0%	100.0%	
Asthma	no	162	189	351	0.087
		46.2%	53.8%	100.0%	
	yes	22	14	36	
		61.1%	38.9%	100.0%	
hypothyroidism	no	162	179	341	0.968
		47.5%	52.5%	100.0%	
	yes	22	24	46	
		47.8%	52.2%	100.0%	
sleep apnea	no	169	195	364	0.080
		46.4%	53.6%	100.0%	
	yes	15	8	23	
		65.2%	34.8%	100.0%	
hiatal hernia	no	167	190	357	0.298
		46.8%	53.2%	100.0%	
	yes	17	13	30	
		56.7%	43.3%	100.0%	
others	no	165	192	357	0.071
		46.2%	53.8%	100.0%	
	yes	19	11	30	
		63.3%	36.7%	100.0%	
age		37.19±10.51	34.68±11.22	35.94±10.95	0.024
BMI before operation		43.74±7.99	44.91±8.12	44.12±8.3	0.157
BMI after operation		28.48±6.43	29.06±6.20	28.7±6.32	0.373

Prevalence of GERD post-surgery and new-onset symptoms

Out of 387 participants, the prevalence of GERD postoperatively was 355 (91.7%), whereas 32 (8.3%) did not develop any GERD-associated symptoms. Approximately 318 (82.2%) developed new-onset symptoms of GERD postoperatively.

## Discussion

The ASMBS initially acknowledged sleeve gastrectomy as an agreeable method for addressing obesity. Moreover, sleeve gastrectomy has been applied as the revision therapy of choice after the failure of most common bariatric procedures [13,14]. Furthermore, LSG has demonstrated positive results for weight reduction and the elimination of associated co-morbidities [15]. Obesity and its co-occurrence with GERD symptoms have been extensively studied and highlighted in clinical investigations [16]. Individuals with GERD may experience significant disruptions to their physical and mental health based on the extent of their symptoms [17]. Multiple factors contribute to reflux symptoms in obese patients. A considerable rise in abdominal pressure leads to lower esophageal sphincter insufficiency, ultimately resulting in GERD [18]. Despite the abundant evidence, the relationship between LSG levels and postoperative GERD symptoms remains unclear. Several studies have reported that LSG exacerbates reflux symptoms compared with other bariatric surgeries, such as Roux-Y gastric bypass, and have recommended that LSG be avoided in patients with preexisting GERD symptoms [19,20]. Other studies, however, opposed the given literature and demonstrated that LSG is not necessarily contraindicated in patients with pre-existing GERD symptoms and contributed to improving their overall well-being and reflux symptoms [21,22]. Hence, conflicting evidence is present on how reflux manifests after LSG. Several hypotheses have been proposed to further define this process while considering technological and anatomical factors. The surgical technique of LSG plays an important role in contributing to postoperative acid reflux, including mobilization of the stomach and left hiatus and alteration of the angle. Another cause is reduced stomach compliance following LSG, which eventually leads to lower esophageal sphincter dysfunction owing to the exposure of the sphincter to high abdominal pressure [23]. In contrast, an accepted belief is widely shared in the literature that LSG may improve GERD symptoms by reducing acid production through resection of the stomach fundus [24]. Furthermore, weight loss reduces intra-abdominal pressure due to reduced visceral fat, which eventually optimizes the function of the lower esophageal sphincter [10]. In this study, 42.0% of patients who suffered from reflux symptoms preoperatively demonstrated complete resolution following LSG. In contrast, Dupree et al. [25] reported that only 16% of their studied patients experienced the resolution of GERD after LSG. Additionally, due to individual differences in esophageal sensitivity, GERD symptoms might be indistinct even in cases of abnormal acid reflux, which could account for the variability in LSG and GERD data [26]. However, a considerable rate (64.6%) of GERD symptoms developed post-LSG. This result is consistent with a study by Dalboh et al. [27], which reported an overall incidence rate of postoperative GERD in their patients (42.3%). Our findings indicated a significant correlation between the occurrence of GERD symptoms after LSG and factors such as being female, having preoperative hyperlipidemia, and being of older age. These findings are consistent with those of Almutairi et al. [28], who showed a significant correlation between age, sex, and GERD onset postoperatively. In contrast, Althuwaini et al. [29] reported that a high preoperative BMI might be indicative of a lower occurrence of heartburn after LSG. Moreover, they reported that preoperative factors could not predict which patients would develop de novo or encounter a deterioration in GERD symptoms following LSG. Deas et al. [22] and Alsuwat et al. [30] reported no significant relationships between age, sex, and BMI and the prevalence of GERD following LSG. We assume that these conflicting results necessitate larger studies that target each possible factor associated with the development of GERD symptoms post-LSG.

This study has some limitations. First, regarding the GERD-HRQL questionnaire used in the study, we translated the questionnaire into Arabic, and despite it being validated in English, no validated Arabic version exists. This can be overcome by conducting a pilot study using a translated version of the questionnaire. In addition, the questionnaire does not measure atypical symptoms of GERD, which increases the risk of missing cases [12]. Our study also examined the possible factors associated with postoperative GERD symptoms. However, factors related to the preoperative state of the patient (except for 69 patients), surgical technique, postoperative complications, or outcomes of the surgery were not accessible due to the study design. Finally, recall bias could have affected our study because a period had passed since the patients underwent surgery.

## Conclusions

In summary, most participants had GERD symptoms after LSG. Furthermore, the development of GERD symptoms after LSG was significantly correlated with age, hyperlipidemia, and sex. Dysphagia, odynophagia, and bloating increased as BMI changed. Similarly, heartburn increased when the BMI decreased. Further research should study the preoperative and surgical factors that could influence the risk of postoperative GERD, add endoscopy and proton pump inhibitors with 24-hour pH monitoring, and determine if a relationship between cultural and lifestyle factors and postoperative outcomes is present.
